# Interstitial Lung Disease in Children With Selected Primary Immunodeficiency Disorders—A Multicenter Observational Study

**DOI:** 10.3389/fimmu.2020.01950

**Published:** 2020-08-27

**Authors:** Małgorzata Pac, Teresa Bielecka, Katarzyna Grzela, Justyna Komarnicka, Renata Langfort, Sylwia Koltan, Nel Dabrowska-Leonik, Katarzyna Bernat-Sitarz, Maciej Pronicki, Hanna Dmenska, Anna Pituch-Noworolska, Bozena Mikoluc, Barbara Piatosa, Katarzyna Tkaczyk, Ewa Bernatowska, Irena Wojsyk-Banaszak, Katarzyna Krenke

**Affiliations:** ^1^Department of Immunology, Children's Memorial Health Institute, Warsaw, Poland; ^2^Department of Pediatric Pneumonology and Allergy, Medical University of Warsaw, Warsaw, Poland; ^3^Department of Radiology, Jan Polikarp Brudziński Pediatric Hospital, Warsaw, Poland; ^4^Department of Radiology, Children's Memorial Health Institute, Warsaw, Poland; ^5^Department of Pathology, National Tuberculosis and Lung Diseases Research Institute, Warsaw, Poland; ^6^Department of Pediatrics, Hematology and Oncology, Collegium Medicum Bydgoszcz, UMK Toruń, Bydgoszcz, Poland; ^7^Department of Pathology, Children's Memorial Health Institute, Warsaw, Poland; ^8^The Pulmonology Outpatient's Clinic, Children's Memorial Health Institute, Warsaw, Poland; ^9^University Children Hospital in Cracow, Medical College, Jagiellonian University, Cracow, Poland; ^10^Department of Pediatrics, Rheumatology, Immunology and Metabolic Bone Diseases, Medical University of Bialystok, Bialystok, Poland; ^11^Histocompatibility Laboratory, Children's Memorial Health Institute (IPCZD), Warsaw, Poland; ^12^Department of Pneumonology, Pediatric Allergology and Clinical Immunology, Poznań University of Medical Sciences, Poznań, Poland

**Keywords:** computed tomography, children, CVID, interstitial lung disease, primary immunodeficiency, GLILD

## Abstract

Primary immunodeficiencies (PIDs) are rare disorders of the immune system encompassing inborn errors of immunity. Primary antibody deficiencies constitute the largest group of PID with common variable immunodeficiency (CVID) being the most common symptomatic form. Combined immunodeficiencies (CID) accompanied by antibody deficiency can mimic CVID and these patients need the verification of the final diagnosis. Respiratory involvement, especially interstitial lung disease (ILD), poses a relevant cause of morbidity and mortality among patients with PID and in some cases is the first manifestation of immunodeficiency. In this study we present a retrospective analysis of a group of children with primary immunodeficiency and ILD - the clinical, radiological, histological characteristics, treatment strategies and outcomes. Eleven children with PID-related ILD were described. The majority of them presented CVID, in three patients CID was recognized. All patients underwent detailed pulmonary diagnostics. In eight of them histological analysis of lung biopsy was performed. We noted that in two out of 11 patients acute onset of ILD with respiratory failure was the first manifestation of the disease and preceded PID diagnosis. The most common histopathological diagnosis was GLILD. Among the analyzed patients three did not require any immunosuppressive therapy. All eight treated children received corticosteroids as initial treatment, but in some of them second-line therapy was introduced. The relevant side effects in some patients were observed. The study demonstrated that the response to corticosteroids is usually prompt. However, the resolution of pulmonary changes may be incomplete and second-line treatment may be necessary.

## Introduction

Primary immunodeficiencies (PIDs) are inherited disorders of the immune system encompassing inborn errors of immunity. The clinical spectrum of PIDs is broad, ranging from relatively mild disorders selectively affecting immune defense mechanisms to serious, life-threatening diseases characterized by profound lack of immune functions. According to a report by the International Union of Immunological Societies (IUIS), over 400 distinct disorders are recognized (1). Estimates by Bousfiha et al. suggest that ~6 million people may be affected worldwide, though only ~60,000 cases of PIDs are currently definitively diagnosed (2). Ten phenotypic categories of PIDs are recognized according to the underlying immune defects (3). Primary antibody deficiencies (PADs) are the largest group of PIDs, accounting for ~55% of PIDs in Europe (www.esid.com) and up to 78% in the USA (4). PADs predominantly result from a primary defect in B cells, but can also be caused by defects in T cells or other immune cell populations that contribute to B-cell or plasma cell development and function. Antibody deficiencies are characterized by a malfunctioning antibody response, which is reflected in low or undetectable levels of immunoglobulin(s).

Common variable immunodeficiency (CVID), with an incidence of 1:25,000–1:50,000, is one of the most prevalent symptomatic PADs (5–7). Some well-defined disorders (e.g., combined immunodeficiencies—CID) accompanied by antibody deficiency can mimic CVID, and only new diagnostic tools, including next-generation sequencing (NGS), enable the verification of the final diagnosis (8, 9).

Patients with antibody deficiency present with a broad spectrum of manifestations. In addition to recurrent infections, non-infectious complications such as interstitial lung disease (ILD), gastrointestinal inflammatory disease, hematologic or organ-specific autoimmunity, lymphoproliferation, and lymphoma may occur (5–8). Respiratory diseases are a cause of morbidity and mortality among PID patients, and in some cases lung disease may play an important role in diagnosis as the first manifestation of PID (10–14). Lung involvement in patients with PID can be infection-related, immune-mediated, or associated with the occurrence of neoplastic diseases. In cases of infection-related lung disease, effective protection can be provided by immunoglobulin replacement therapy (IgRT); however, it does not prevent non-infectious pulmonary involvement (6, 12). ILDs represent one of the most significant immune-mediated complications of PID (14). The most common form of PID-related ILD is granulomatous-lymphocytic ILD (GLILD), an umbrella term that encompasses a spectrum of lung pathologies: various forms of pulmonary lymphoid hyperplasia [lymphocytic interstitial pneumonia (LIP), follicular bronchiolitis (FB), nodular lymphoid hyperplasia, and granulomatous disease], as well as organizing pneumonia (10, 14, 15). The risk of death in CVID patients with non-infectious complications like ILD has been shown to be several times higher than in patients with infectious complications only (16). CVID-related GLILD significantly shortens life expectancy (17).

Previous reports concerning PID-related ILD are mainly limited to adults; data regarding disease specificity and exceptionality of pediatric patients available in the literature are insufficient. Herein we present a retrospective analysis of the clinical, radiological, and histological characteristics, as well as treatment strategies and outcomes, in a unique group of children with PID-related ILD, predominantly CVID patients.

## Materials and Methods

Retrospective analysis of clinical data from children with PID-related ILD consulted or treated consecutively in the Department of Immunology at the Children's Memorial Health Institute (CMHI) of Warsaw and the Department of Pediatric Pneumonology and Allergy at the Medical University of Warsaw between 2012 and 2019 is presented.

The major inclusion criterion was the diagnosis of PID, mostly CVID. Diagnosis of CVID was established according to ESID diagnostic criteria (18). Since CVID encompasses a group of heterogenous antibody deficiencies with many monogenic forms, detailed immunophenotyping and genetic studies using next-generation sequencing (NGS), Sanger sequencing, or fluorescence *in situ* hybridization (FISH) were performed in six individuals to better characterize the study group. In five patients genetic tests were not performed because of: technical problems with genetic testing and consent not given by parents. Following laboratory and/or molecular studies, combined immunodeficiency (CID) was diagnosed in three individuals: one with Nijmegen breakage syndrome (NBS), one with DiGeorge Syndrome (DGS), and one with LRBA.

Diagnosis of PID-related ILD was based on (1) clinical signs and symptoms [tachypnea [defined as respiratory rate >90th percentile (19)], retractions, crackles]; (2) typical computed tomography (CT) findings [multiple nodules of different densities with perilymphatic or interlobular distribution predominantly in the middle and lower zones of the lungs and hilar or mediastinal lymphadenopathy (transverse dimension of the lymph nodes >1 cm)]; and (3) histopathology of lung biopsy with findings consistent with GLILD, cryptogenic organizing pneumonia (COP), LIP, FB, or lymphoid hyperplasia.

Exclusion criteria were other ILDs, coexistence of other chronic lung diseases (e.g., cystic fibrosis), and acute or chronic respiratory infections.

### Data Collection

Prespecified data on all children with PID and related ILD were collected. These included (1) demographic data; (2) clinical signs and symptoms such as dyspnea, tachypnea, cough, cyanosis, crackles, wheezing, respiratory failure, finger clubbing, splenomegaly, hepatomegaly, lymphadenopathy, hemolytic anemia, thrombocytopenia, and history of chronic lung diseases; (3) the results of the following laboratory tests: total blood count, detailed immunological screening (including immunoglobulin level and immunophenotyping of lymphocytes, with B- and T-cell functional tests and genetic testing in some cases); (4) the results of pulmonary function tests, including spirometry, body plethysmography, and diffusing capacity for carbon monoxide (DL_CO_); (5) the results of lung high-resolution computed tomography (HRCT) and chest magnetic resonance imaging (MRI); (6) histopathology of lung biopsy; and (7) treatment regimens [corticosteroids, other immunosuppressive drugs (e.g., azathioprine, mycophenolate mofetil, rapamycin, cyclosporin), IgRT, and hematopoietic stem cell transplantation (HSCT)], duration, and outcomes.

The immune status of patients was assessed based on serum immunoglobulin concentration, antibody response to vaccines and/or isohemagglutinins titer, immunophenotyping of lymphocytes with switch memory B cells numbers, and lymphoproliferation tests as described previously [(20–22); data not shown]. The sources of data were an electronic database of patients with PID and medical records of patients with PID-related ILD.

### Imaging Studies

In eight children, volumetric CT examinations were performed with either a 320-row multidetector computed tomography scanner (Aquilion ONE, Toshiba, Japan; three children) or a 128-row multidetector computed tomography scanner (SOMATOM Definition AS, Siemens, Germany; five children) and images were reconstructed using a high-resolution reconstruction algorithm. In the remaining two patients, CT scans performed in local hospitals were used for analysis. Contrast medium was administered in eight patients. Pulmonary involvement in the child with NBS with a known radiosensitivity was monitored with chest MRI.

### Pulmonary Function Tests

Spirometry tests were obtained in nine cooperating individuals and included forced vital capacity (FVC) and forced expiratory volume in 1 second (FEV1). All pulmonary function tests were performed in the sitting position using a spirometer (Lungtest 1000 MES, Poland, or JAEGER MasterScreen, Germany) according to the ERS (European Respiratory Society) guidelines for lung function testing (23). All spirometric results were presented as percentiles (pc). FVC, FEV1, and FEV1/FVC ≥5th pc were considered normal. Obstruction was diagnosed if the FEV1/FVC ratio was below the 5th pc. DL_CO_ was measured using the single-breath method using a Vmax22 spirometer (SensorMedics, USA), or MasterScreen (CareFusion, USA) and results between the 5th and 95th pc were considered to be within the normal range. The whole-body plethysmography was performed using a Lungtest 1000 (MES, Poland), or MasterScreen (CareFusion, USA). Total lung capacity (TLC) and residual volume (RV) were calculated for each patient. TLC lower than the 5th pc was considered to indicate restriction. Air trapping was defined as a RV/TLC ratio above the 95th pc.

### Histological Assessment

Lung biopsies were performed in eight of the 11 patients. The obtained material was then fixed in 10% buffered formalin solution and paraffin embedded (FFPE). Microscopic slides with 4-μm FFPE sections were stained with hematoxylin and eosin (H + E) and in some cases immunohistochemical markers were used (CD3, CD20, CD5, CKAE1/AE3, and Ki-67; Ventana Roche, USA). Immunohistochemical tests were performed using an automated Ventana Benchmark GX Slide Staining System according to the manufacturer's protocol. In two cases, mediastinal lymph nodes biopsies were analyzed.

### Statistical Analysis

The results of the study were summarized using standard descriptive statistics. Data are presented as median and range.

The studies involving human participants were reviewed and approved by The Bioethics Committee of the Children's Memorial Health Institute in Warsaw (approval no.9/KBE/2020). All parents or legal guardians, as well as patients older than 16 years of age, gave their written consent to participate in the study.

## Results

### Demographic and Clinical Data

Eleven children with interstitial lung disease were selected from the database of 796 children with PID followed-up between 2012 and 2019. Detailed immunological and genetic diagnostics enabled the diagnosis of CVID in nine patients however, in one of these patients, finally an NGS panel revealed an *LRBA* mutation. The other two patients with a provisional diagnosis of PAD were eventually diagnosed with CID with associated features (NBS in the patient with hydrocephaly and DGS in the patient without an expressive dysmorphia). In three patients with CVID no mutations were confirmed.

All of the children diagnosed with PID-related ILD were boys.

The median age at the diagnosis of antibody deficiency was 9.5 years (range 4–17 years). Six patients had never developed respiratory symptoms (except respiratory tract infections). Chest imaging studies were performed for other reasons (e.g., generalized lymphadenopathy) and revealed pulmonary involvement. Five patients were symptomatic in terms of respiratory involvement. In two children, the symptoms of ILD preceded the recognition of immunodeficiency, so the median age at the onset of ILD symptoms was 6.5 years (range 2–16). The severity of ILD symptoms was diverse, with one child presenting with a mild cough (patient No. 4; Table 1) and three others with life-threatening respiratory failure (patients No. 3, 7, and 8).

**Table 1:** Baseline clinical characteristics at diagnosis.

**Patient**	**1**	**2**	**3**	**4**	**5**	**6**	**7**	**8**	**9**	**10**	**11**
Age, sex	13, M	19, M	13, M	19, M	5, M	16, M	10, M	12, M	11, M	18, M	17, M
Age at symptom onset	4 yr	11 yr	11 yr	3 yr	3 yr	16 yr	2 yr	8 yr	5 yr	10 yr	9 yr
Presenting symptoms	Splenomegaly, thrombocyte-penia,	Pain in the lower limbs, lymphadenopathy, splenomegaly, anemia, leucopenia	Dyspnea, crackles, acute respiratory failure	Generalized lymphadenopathy	Skin nodules, generalized lymphadenopathy, hepatospleno-megaly, pancytopenia	Hepatospleno-megaly, anemia, lymphadenopathy	Dyspnea, crackles, acute respiratory failure	Severe viral enterocolitis, parakeratosis, facial dysmorphia, hydrocephalus, strabismus, hypospadias, cryptorchidism	Hepatospleno-megaly, pancytopenia	Lymphadeno-pathy, hepatospleno-megaly	Recurrent pneumonia, lymphadeno-pathy
Recurrent respiratory infections	+	–	–	–	–	–	–	–	+	+	+
Age at ILD diagnosis	8 yr	17 yr	11 yr	13 yr	4 yr	16 yr	2.5 yr	10 yr	9 yr	10 yr	14 yr
Age at PID diagnosis	5 yr	17 yr	12 yr	12 yr	4 yr	16 yr	7 yr	4 yr	5 yr	15 yr	14 yr
Immunodeficiency	CVID	CVID	CVID	CVID	LRBA	CVID	CVID	NBS	CVID	DGS	CVID
Genetic testing	–	–	–	+ No changes	+ LRBA	–	+ No changes	+ NBS	+ No changes	+ DGS	–
**ILD manifestation:**											
Chronic cough	+	–	–	+	–	–	–	+	–	–	–
Dyspnea	–	–	+	–	–	–	+	+	–	–	–
Crackles	+	–	+	+	–	–	+	+	–	–	–
Clubbed fingers	–	–	–	–	–	–	+	–	–	–	–
Acute respiratory insufficiency	–	–	+	–	–	–	+	+	–	–	–
**Extra-pulmonary manifestation:**											
Hepatomegaly	+	+	+	–	+	+	+	–	+	+	–
Splenomegaly	–	+	+	–	+	+	+	–	+	+	_
Lymphadenopathy	+	+	–	+	+	+	–	–	+	+	+
**Cytopenia:**											
Anemia	–	+	–	–	+	+	+	–	–	–	–
Leukopenia	–	+	–	–	+	+	–	–	+	–	–
Thrombocytopenia	+	–	–	–	+	+	+	–	+	–	–
Lung HRCT findings^*^:											
Nodules	+	+	+	+	+	+	+	+	+	+	+
Perilymphatic distribution	+	+	+	+	+	+	–	–	–	+	+
Middle and lower zone predominance	+	+	+	+	–	+	–	+	–	+	–
Intrathoracic lymhadenopathy	+	–	–	+	+	–	+	+	–	+	+
Ground-glass opacities	–	–	+	+	–	+	+	–	–	–	–
Lung consolidations	+	–	+	+	+	–	+	–	–	–	+
Lungbiopsyresult	GLILD/LIP	GLILD/LIP	GLILD	GLILD/LIP	GLILD/LIP	GLILD	GLILD/LIP	Granulomas	–	–	–
Lymph node biopsy result						Granulomas				Granulomas	

* *Patient 8 had only chest X-ray and chest MRI done. Due to increased risk of cancer after radiation exposure, he was disqualified from chest CT*.

*PID, primary immunodeficiency; CVID, common variable immunodeficiency; LRBA, mutation in the lipopolysaccharide-responsive and beige-like anchor protein (LRBA) gene; NBS, Nijmegen breakage syndrome; ILD, interstitial lung disease; HRCT, high resolution chest tomography; GLILD, granulomatous-lymphocytic interstitial lung disease; FB, follicular bronchiolitis; LIP, lymphocytic interstitial pneumonia; COP, cryptogenic organizing pneumonia*.

Detailed demographic data and clinical symptoms are presented in Table 1.

### Computed Tomography

Lung CT was performed in 10 patients. One child with NBS had been diagnosed and followed with chest MRI to avoid radiation exposure.

**Figure 1:**
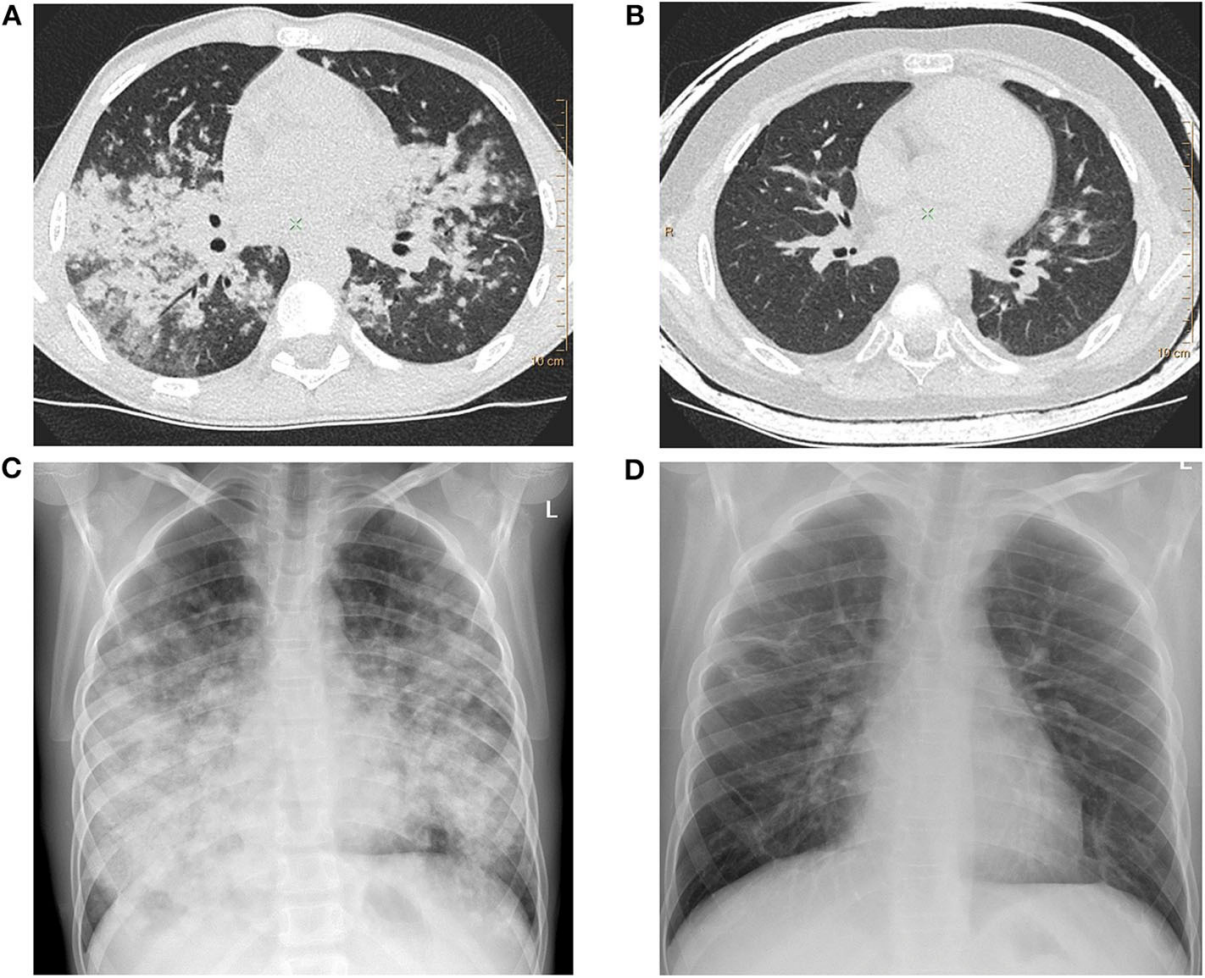
**(A)** Lung high-resolution computed tomography (HRCT): multiple, poorly defined nodules merging in larger areas of ground-glass opacities and consolidations. **(B)** Lung HRCT: complete regression of pulmonary changes; residual irregular linear opacities are visible. **(C)** Chest X-ray: diffuse poorly defined nodules creating larger areas of lung consolidations with adjacent areas of ground-glass opacities. Middle and lower zone predominance is clearly marked. **(D)** Chest X-ray: complete resolution of pulmonary opacities with residual irregular linear thickenings.

**Figure 2:**
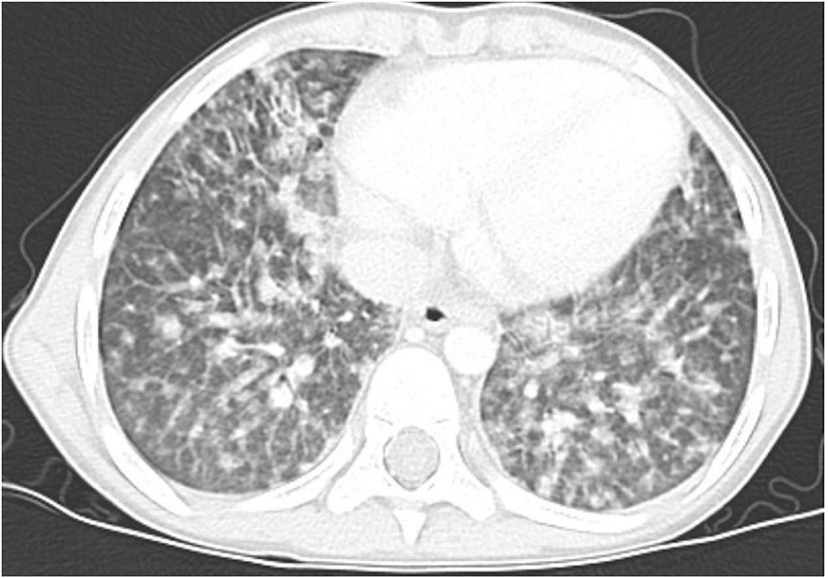
Lung high-resolution computed tomography: multiple ground-glass and solid nodules scattered across the lungs, with perylimphatic distribution. Larger clusters of nodules and tree-in-bud pattern are visible peripherally.

**Figure 3:**
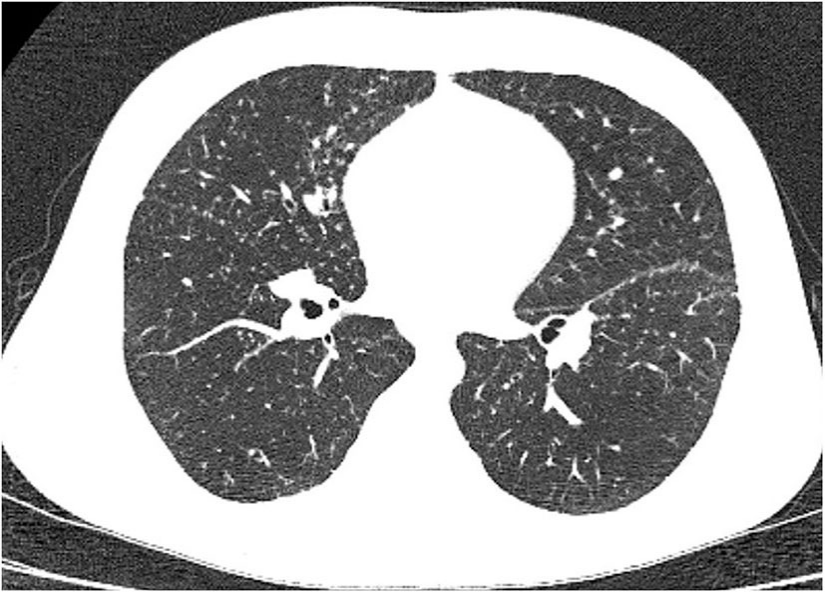
Lung high-resolution computed tomography: multiple small pulmonary nodules with perilymphatic distribution (clearly seen along pleural fissures).

**Figure 4:**
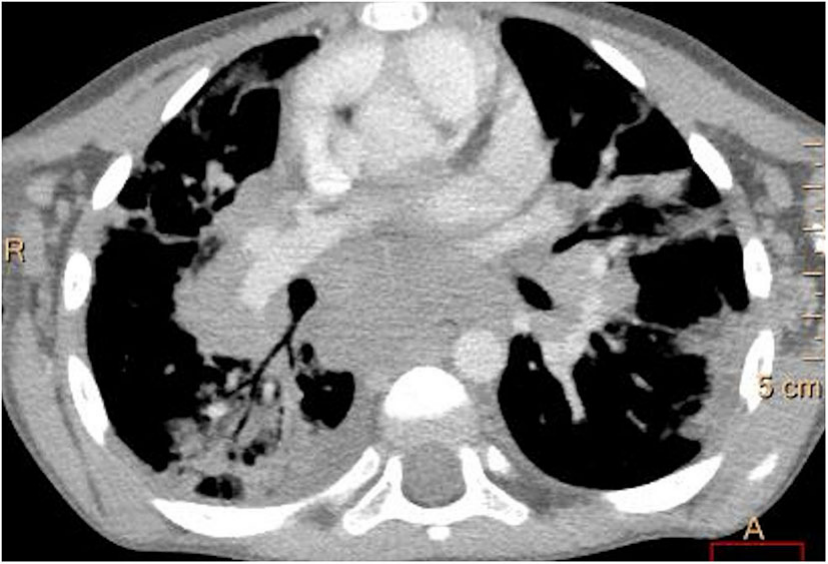
Contrast-enhanced lung computed tomography (soft tissue window): marked enlargement of mediastinal and hilar lymph nodes with areas of subpleural lung consolidations.

Multiple, mostly poorly defined pulmonary nodules were revealed in all children assessed. The diameter of the nodules ranged from 2 to 30 mm and their density varied from solid to part-solid to ground glass opacity (Figures 1A, 2, 3). Solid nodules were surrounded by a ground-glass halo in four patients. In most of the children, nodules had a perilymphatic distribution with a predilection for the middle and lower lung zones. The lesions formed rosettes and a “tree-in-bud” pattern in one patient (Figure 2). Interlobular septal thickenings or irregular linear opacities were found in one case each. The changes described above were accompanied by hilar and/or mediastinal lymphadenopathy in seven patients (Figure 4).

During the exacerbations of ILD, progression of lung involvement in imaging studies was observed, with an increase in the number, size, and density of lung nodules as well as extensive areas of ground-glass opacities and parenchymal lung consolidations (Figures 1A,C).

### Pulmonary Function Tests

Pulmonary function tests (PFTs) were performed in nine children. Two children were unable to perform PFT, one due to the young age and the other due to intellectual disability. All of the PFTs were performed in the stable phase of the ILD. The results of PFT are shown in Table 2. We found one patient (No. 3) with a restrictive ventilatory pattern and five with air trapping (No. 2, 4, 9, 10, and 11). DL_CO_ was abnormal in two patients. A mild reduction of DL_CO_ was found in patient No. 2 (74% of predicted value) and a moderate reduction was observed in patient No. 3 (50% of predicted value).

**Table 2:** Results of pulmonary function tests.

**Pulmonary function tests**	**Patient**	**1**	**2**	**3**	4^*^	**5**	**6**	7^*^	**8**	**9**	**10**	**11**
Spirometry	**FEV1**, percentile	94	15.64	0.03	11.39	ND	75	22	ND	29.96	25.43	97.03
	**FVC**; percentile	10	18.50	0.02	7.08	ND	50	13	ND	48.97	13.91	80.49
	**FEV1/FVC**; percentile	38	36.29	84.84	46.88	ND	70	80	ND	20.11	60.37	94.58
Plethysmography	**RV**; percentile	ND	100	0.07	99.98	ND	97	ND	ND	100	100	100
	**TLC**; percentile	ND	99.96	0.0	90.35	ND	>99	ND	ND	99.82	98.94	99.97
	**RV/TLC**; percentile	ND	99.91	0.78	99.62	ND	73	ND	ND	99.94	99.82	99.17
Diffusing capacity for carbon monoxide	**DLCO**; percentile	ND	0	0	55.08	ND	41	ND	ND	97.39	90.13	ND

* *Lung function tests were performed in a stable phase of the disease, during treatment*.

*ND, No data*.

*DLCO, diffusing capacity for carbon monoxide; FEV1, forced expiratory volume in 1 second; FVC, forced vital capacity; RV, residual volume; TLC, total lung capacity*.

The results of the PFTs are presented in Table 2.

### Lung Biopsy Results

**Figure 5:**
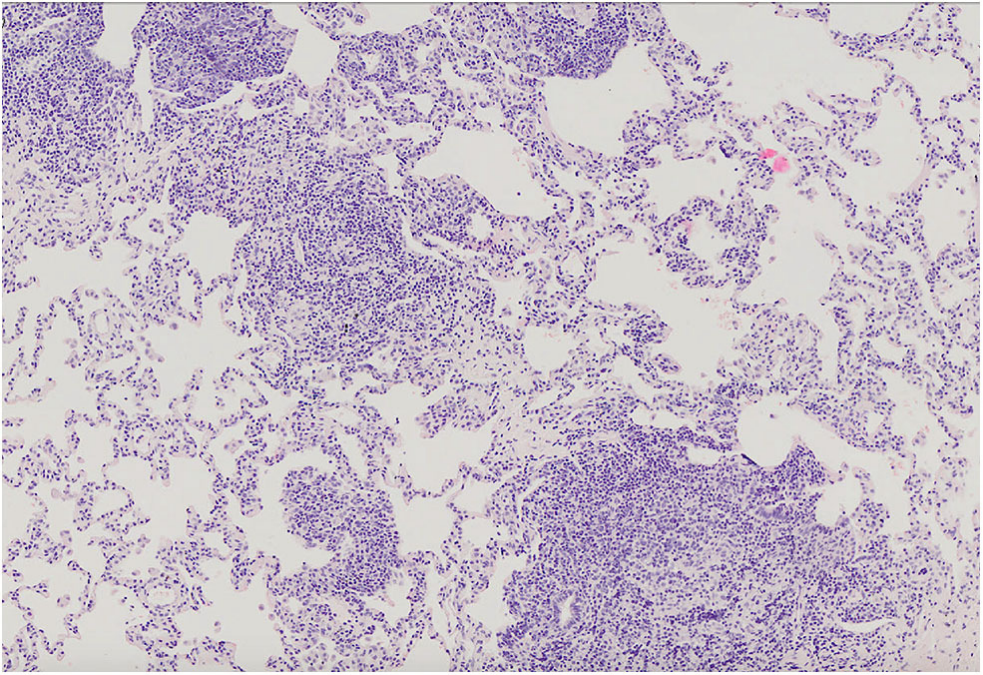
Lung parenchyma with diffuse interstitial and peribronchiolar lymphocytic infiltration. Microphotograph: hematoxylin and eosin stain.

**Figure 6:**
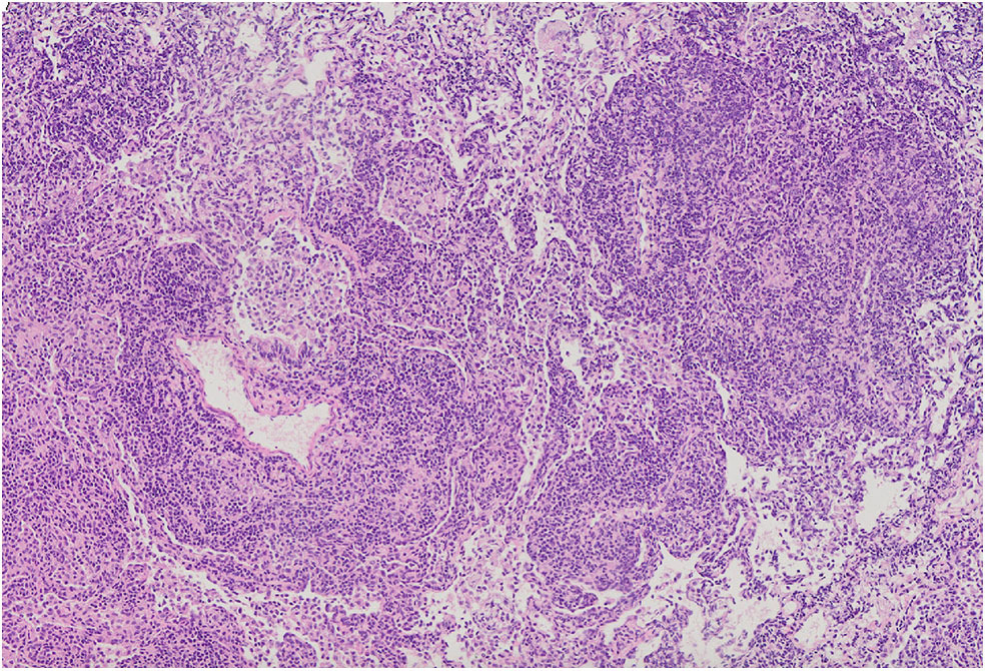
Diffuse intensive lymphocytic infiltration, both interstitial and around blood vessels. Alveolar spaces are filled with numerous macrophages. Microphotograph: hematoxylin and eosin stain.

The most common histopathological diagnosis was GLILD (seven cases), with a predominance of the LIP pattern (five cases; Figure 5). Microscopic features of LIP were dominated by abundant diffuse interstitial infiltrates of lymphoid cells composed of CD3+ and CD20+ lymphocytes. In some patients, the inflammatory infiltration formed clusters with single, small lymph nodules. In addition to the intensive interstitial infiltration, a marked peribronchial and perivascular chronic inflammation was present (Figure 6).

**Figure 7:**
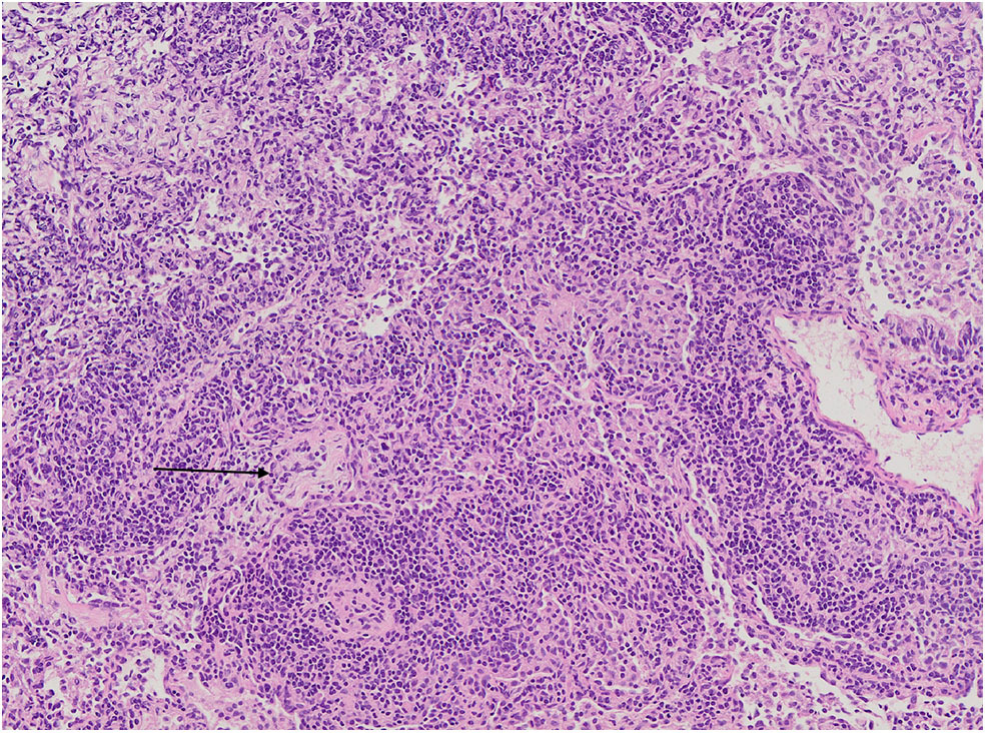
Diffuse interstitial inflammation with small focus of organizing pneumonia (black arrow). The surrounding alveoli are filled with numerous macrophages. Microphotograph: hematoxylin and eosin stain.

Foci of COP and FB were found in addition to LIP features in one of the patients (Figure 7).

Interstitial lymphocytic infiltrates were accompanied by the presence of multiple non-necrotizing granulomas in two children.

In the child diagnosed with NBS, the only histopathological abnormality in the lung biopsy was non-necrotizing granulomas.

In two patients (No. 6 and 10), a mediastinal lymph node biopsy was performed and revealed non-caseating granulomas.

### Treatment Strategies

In eight children with PID-related ILD, the immunosuppressive treatment was introduced (No. 1–8). In three patients (No. 3, 7, and 8) severe pulmonary symptoms were the only reason for initiating the treatment. Patients 1, 4, 5, and 6 (one symptomatic, three asymptomatic) were treated due to autoimmune cytopenia and/or lymphoproliferation with coexisting extensive lung involvement. Patient No. 2, who didn't present with respiratory symptoms, was qualified for treatment because of decreased DL_CO_ results, progression of lung infiltrates in chest HRCT and features of fibrosis in the lung biopsy.

Three patients, due to a lack of clinical symptoms and stable, low-intensity changes in the lung HRCT, did not qualify for any immunosuppressive therapy (No. 9, 10, and 11).

Systemic glucocorticoids (GCs) were introduced in all eight children at the onset of treatment. Two children were initially treated with corticosteroid pulses. GCs were the only drugs used in four patients (No. 2, 4, 6, and 7). In one of the patients (No. 4), initially a recurrence of symptoms was observed during GC dose reduction. It is worth noting that despite the treatment discontinuation after the patient's decision at the age of 18, he remains clinically and radiologically stable. In patient No. 7, a recurrence of ILD occurred after an asymptomatic period of several months. Patient No. 6 (clinically asymptomatic) started treatment with GCs due to autoimmune hemolytic anemia. Hematological improvement after 3 months of GC therapy was achieved, without resolution of radiological findings.

GC treatment resulted in rapid improvement of general condition and resolution of respiratory failure in patient No. 3. However, exercise intolerance, significant radiological abnormalities, and abnormal PFT results persisted despite the therapy.

Two boys were additionally treated with azathioprine (No. 1 and 3), achieving a meaningful clinical improvement, but due to serious side effects of the therapy (Table 3), the treatment was discontinued. In patient No. 1, mycophenolate mofetil was introduced, but the treatment was ineffective (thrombocytopenia did not resolve) so the boy was eventually qualified for HSCT. The post-transplant period was complicated by infections caused by HHV-6, *Pneumocystis jiroveci* and *Streptococcus viridans* shortly after transplantation. After HSCT a resolution of respiratory symptoms and regression of pulmonary abnormalities in imaging studies were achieved. Some months after the transplantation, due to the refractory thrombocytopenia, splenectomy was performed with a satisfactory effect. Despite the discontinuation of azathioprine, patient No. 3 maintained the significant clinical and radiological improvement obtained on the therapy (Figures 1B,D). In patient No. 8, the resolution of ILD symptoms was achieved after adding cyclosporin to GCs.

**Table 3:** Treatment strategies.

**Patient (lung biopsy)**	**Treatment line**	**Treatment effect**	**Treatment complications**	**Comments**
1(GLILD/LIP)	First Line: DEX 1.0 mg/kg 10 days, PRED, initial dose: 1.0 mg/kg, maintenance dose: 0.2 mg/kg	Clinical and radiological improvement, but persistence of residual lesions in lung HRCT	Cushing syndrome	Introduction of second-line treatment
	Second Line (1): AZA 2mg/kg	Clinical stabilization	Pancreatitis	Due to pancreatitis, AZA was changed to MMF
	Second Line (2): MMF 500 mg/day	Clinical and radiological progression	Progression of lung infiltrates, persistent lymphadenopathy, splenomegaly, leukopenia, and hypogammaglobulinemia on a low dose of steroids and MMF	Patient qualified for HSCT, infectious complications after HSCT (HHV-6, Pneumocystis jiroveci, Str. viridans), splenectomy due to refractory thrombocytopenia and finally clinical and radiological improvement
2 (GLILD/LIP)	PRED initial dose: 0.75 mg/kg, maintenance dose: 0.33 mg/kg for 6 months	Radiological improvement	–	Patient had no clinical respiratory symptoms
3 (GLILD)	First Line: MPRED i.v. pulses 3 days (30 mg/kg), PRED initial dose: 2.0 mg/kg, maintenance dose: 0.4 mg/kg	Resolution of respiratory failure, incomplete clinical (exercise intolerance) and radiological (partial resolution of nodular lesions) improvement	Cushing syndrome, osteoporosis with multiple compression fractures of lumbar vertebrae	Clinical and radiological deterioration observed while reducing the steroid dose; therefore, AZA was introduced
	Second Line: AZA 2.0–3.5 mg/kg	Significant clinical and radiological improvement	Hepatitis with liver injury and cholestasis	AZA dose was modified depending on the 6-thioguanine levels; due to adverse effects, treatment was discontinued
4 (GLILD/LIP)	PRED initial dose: 2.0 mg/kg, maintenance dose: 0.6 mg/kg	Resolution of clinical symptoms, partial radiological improvement	Cushing syndrome	Recurrence of radiological changes while trying to discontinue the drug
5 (GLILD/LIP)	First Line: PRED initial dose: 2.0 mg/kg, maintenance treatment: MPRED 0.1 mg/kg in tapered doses	Almost total resolution of radiological changes	Severe steroid-dependent thrombocytopenia	
	Second Line (1): MMF 400 mg/day	Almost total resolution of radiological changes		MMF was introduced almost simultaneously with PRED
	Second Line (2): RAP 1.6–1.4 mg/kg		Transient leucopenia, hypertransaminasemia	Due to thrombocytopenia, MMF was replaced with RAP
6 (GLILD)	PRED initial dose: 0.7 mg/kg, maintenance dose: 0.5 mg/kg	Without radiological improvement	Cushing syndrome, depression	The patient reports periodically a subjective feeling of dyspnea
7 (GLILD/LIP)	PRED initial dose: 2.0 mg/kg, maintenance dose 0.2 mg/kg	Significant clinical and radiological improvement	Cushing syndrome	
8 (G)	First Line: PRED Initial dose: 1.0 mg/kg, maintenance dose: 0.2 mg/kg	Clinical and radiological improvement	Cushing syndrome	
	Second Line: CsA 2.5–3.0 mg/kg	Clinical stabilization		

*AZA, azathioprine; CsA, cyclosporine A; DEX, dexamethasone; G, granulomas; GLILD, granulomatous-lymphocytic interstitial lung disease; HSCT, hematopoietic stem cell transplantation; HRCT, high-resolution chest tomography; LIP, lymphocytic interstitial pneumonia; MMF, mycophenolate mofetil; MPRED, Methylprednisolone; PRED, prednisone; RAP, rapamycin*.

All of the patients remain alive. A clinical and radiological improvement or stability of the lung disease is observed in all the patients.

Significant side effects were observed during the immunosuppressive treatment. Six children treated with GCs presented with symptoms of Cushing syndrome. In patient No. 3, compression fractures of the lumbar vertebrae occurred due to post-steroid osteoporosis.

The azathioprine treatment was complicated by pancreatitis in patient No. 1 and by liver damage with cholestasis in patient No. 3. In both patients, signs of drug-induced organ damage resolved after discontinuation of the treatment. Transient cytopenia and elevated transaminase levels were observed during the treatment with rapamycin (second-line therapy, patient No. 5). In this case, almost total resolution of radiological changes was observed.

All of the children were receiving IgRT during the study.

The treatment strategies are presented in Table 3.

## Discussion

Clinical presentations of PIDs are diverse, and PIDs are often a diagnostic challenge. This is due in part to pulmonary changes in patients with PIDs, as these changes may have different etiologies and varied clinical as well as radiological presentations, including ILD. Although our study showed a low prevalence of PID-related ILD in children, it highlights various clinical courses of ILDs, including life-threatening complications. Importantly, the study demonstrated that ILD may precede PID diagnosis and that the response to corticosteroids is usually prompt. However, the resolution of pulmonary changes may be incomplete and a second-line treatment may be necessary in some patients. It should be highlighted that in 3 patients, the initial symptoms suggested CVID. However, further observation and the occurrence of other symptoms, including those suggestive of ILD, led to an in-depth diagnostics and the final diagnoses were established based on genetic testing. The achieved diagnoses resulted in changing both, the diagnostic (e.g., NBS) and therapeutic (e.g., DGS and LRBA) management. We would like to emphasize that this is the largest case series on PID-related ILDs ever reported in a pediatric population. Thus, we believe our findings may add to the existing literature on the topic.

GLILD was originally described in patients with CVID (17). Currently, there is a growing number of studies reporting this disease in other PIDs, including DGS and LRBA deficiency (24–26). Moreover, interstitial lymphocytic lung disease was recognized in 4 NBS patients (11%) described by Deripapa et al. (27). However, in the largest group of NBS patients, published by Wolska-Kuśnierz et al., infections clearly dominated among pulmonary complications (28).

The clinical picture of PID-related ILD in our study was very diverse. It should be stressed that in two of the 11 children (No. 3 and 7), ILD was the first presentation, documented even before PID diagnosis. Similar sequences of symptoms and diagnosis were recently reported in adults (29). Importantly, in both children, the presenting symptoms were severe and progressed to respiratory failure. The first child had four episodes of ILD exacerbations with respiratory failure and biopsy-proven LIP diagnosis 3 years before CVID diagnosis. In the second child, pulmonary signs and symptoms, which were initially interpreted as pneumonia, deteriorated rapidly to respiratory failure (Figures 1A,C). The clinical picture, imaging studies, and lung biopsy were consistent with GLILD, but the definitive diagnosis of CVID was established only 7 months later. Respiratory failure also occurred in the other child, but in contrast to the patients mentioned above, ILD symptoms appeared 3 years after the diagnosis of NBS.

In contrast to the three patients with severe lung disease, the majority of children with PID-related ILD were asymptomatic or presented mild respiratory symptoms, but had extensive pulmonary involvement in imaging studies. Thus, we strongly agree with the British Lung Foundation/United Kingdom Primary Immunodeficiency Network Consensus (BLF/UKPINC) that patients with CVID should be screened for pulmonary complications despite the absence of symptoms. HRCT, lung function tests, and other diagnostic procedures are recommended as a part of the diagnostic workup (30). Regular clinical monitoring for lung involvement should also apply to patients with other PIDs (31).

In addition to respiratory symptoms, patients with PIDs present with a wide spectrum of symptoms involving other organs. In a significant number of patients, autoimmune cytopenias, especially autoimmune thrombocytopenia, are observed as the first manifestation of the disease (32). This was the case of two our patients with autoimmune cytopenia, in whom autoimmune cytopenia was the first presenting symptom. Quinti et al. described autoimmune cytopenia as the only complication in 2.3% of CVID patients and autoimmune phenomena as the most common complication [17%; (33)]. Lymphadenopathy, hepatomegaly, and splenomegaly were the most common presentations in our study group. The other non-infectious manifestations included chronic diarrhea, allergic diseases, lymphoid hyperplasia, and failure to thrive.

GLILD can be also found as a serious complication of PIDs in adults. As in children, the clinical course of PID-related ILD in adults can be asymptomatic. Symptomatic patients usually present with dyspnea, tachypnea, cough, exercise intolerance of various severity, and crepitations. Similar to children, hypoxemic respiratory failure may occur in the most severe cases (17, 29, 34).

According to the BLF/UKPINC, the most typical radiologic findings in GLILD are solid or semisolid nodules, ground-glass opacities, and enlarged thoracic lymph nodes (30). These data are consistent with our observations. Nodules of different sizes and densities were found in all children, with perilymphatic distribution (along pleura, fissures, interlobular septa, and bronchovascular bundles) predominating in eight out of 11 patients. A similar distribution of nodules was previously observed in patients with CVID-related ILD (35–38). However, other radiographic patterns were also reported, including random distribution (35–37, 39, 40) and mixed distribution with randomly scattered small nodules (<5 mm) and peribronchial localization of larger nodules (39).

Ground-glass opacities were identified in four patients with PID-related ILD. In GLILD, patchy areas of ground-glass opacities are usually located peripherally and in peribronchial and subpleural regions. These changes may reflect organizing pneumonia areas (10, 35, 39, 41, 42). “Halo sign” was found in two patients in our study group. Bouvry et al. reported that this sign was a common feature of GLILD and was associated with the presence of granulomas, which may be surrounded by foci of organizing pneumonia (37).

In two of the children in this study, CT revealed a tree-in-bud pattern. This pattern may reflect a spectrum of endo- and peribronchiolar disorders, including mucoid impaction. Tree-in-bud sign was reported by Bang et al. in CVID patients with pulmonary infections (43). This sign may also reflect non-infectious peribronchial or bronchiolar inflammation and was found in patients with follicular bronchiolitis (44) or, less commonly, in organizing pneumonia (44, 45).

In our patients, lung HRCT did not reveal reticular abnormalities, which appear to be common features of GLILD in adults (17, 39–41, 46). As reticular opacities seem to be features of more advanced stages of ILD (47), we propose that the presence of a reticular pattern in adults is associated with more prolonged lung involvement resulting in irreversible fibrotic changes in the lung architecture.

The data on pulmonary function in patients with PID-related ILDs mainly come from adult studies. Restrictive or mixed obstructive and restrictive patterns, as well as decreased DL_CO_, were the most frequently observed abnormalities. However, it must be underlined that the results of PFT can be completely normal in the majority of patients (14, 35, 43). Similar observations were made in the very few studies on children. A restrictive pattern was reported by Tillman et al. in a 13-year-old girl with GLILD and by van de Ven et al. in eight children with CVID and CVID-like disease (48, 49). In our study, restrictive pulmonary dysfunction was demonstrated in one child (patient No. 3). Interestingly, an increased RV/TLC ratio indicating air trapping in the absence of FEV1/FVC reduction was the most common finding in our study. According to some authors, an increased RV/TLC ratio may be a more sensitive indicator of airflow obstruction than the FEV1/FVC ratio (50, 51). This parameter is thought to reflect distal airway narrowing or closure resulting from inflammation (50, 51). As nodular peribronchiolar lymphocytic inflammation is common in CVID patients with GLILD (52), we can hypothesize that our findings reflect small airway obstruction caused by an increased number of lymphocytes in this region. Decreased DL_CO_, which is an early indicator of ILD (35, 36), was noted in two of our patients (No. 2 and 3).

In the light of data demonstrating that GLILD is associated with significantly increased mortality in patients with CVID, identifying an effective treatment is a critical issue (16). Although no standard treatment has been established, the consensus statement of the BLF/UKPINC (30) recommends optimizing IgRT as an initial step in the treatment of CVID. However, despite adequate IgRT, complications like CVID-related ILD may occur in some patients, making additional therapy necessary.

According to the aforementioned consensus statement, systemic corticosteroids should be applied as the first-line therapy in symptomatic patients. In our group, GCs were administered to all eight patients who required an intervention in addition to IgRT. In four patients decision about treatment was made due to the coexistence of autoimmune cytopenia or lymphoproliferation and extensive lung involvement in imaging studies. In patient No. 2 the treatment with GCs was started owing to significant progression in imaging studies, decreasing DL_CO_, as well histopathological features of irreversible lung scarring. The use of GCs as monotherapy led to rapid improvement in almost all the treated individuals. Their effectiveness was particularly evident in patients with the most severe cases of ILD (No. 3, 7, and 8), whose respiratory failure resolved in response to the treatment. However, in the four patients who did not fully respond to the first-line therapy, developed significant side effects, or presented with disease exacerbation during steroid tapering, second-line treatment was applied. The second-line treatment drugs that were effective in our group were mycophenolate mofetil, azathioprine, cyclosporin A and rapamycin. These agents improved the clinical course of GLILD in three of our patients. However, it should be stressed that immunosuppressive treatment was associated with several significant side effects. Among the most relevant were: leucopenia after MMF treatment; pancreatitis, liver injury with hypertransaminasemia and cholestasis after azathioprine therapy; leucopenia and hypertransaminasemia after rapamycin treatment and advanced osteoporosis with compression fractures of lumbar vertebrae after GCs therapy (Table 3). In our study, we used either GCs alone (four patients) or a combination of immune-modulating agents (four patients). Since the second-line drugs rapamycin and azathioprine are directed toward T cells, they are effective in GLILD patients, whose lung biopsies are known to contain infiltrates with T cells. We found that this type of therapy brought about subjective and objective resolution of ILD in PAD patients. As reported by other authors, similar effects may be obtained with the CTLA-4 fusion protein abatacept. The effectiveness of this treatment has been documented, particularly in patients with LRBA deficiency (53–55). Our patient with LRBA deficiency was treated with GCs as a first-line therapy, and mycophenolate mofetil and rapamycin as a second-line therapy, but did not receive abatacept treatment.

As lung infiltrates also consist of CD20+ B cells, clinical improvement in GLILD may also be achieved after administration of the anti-CD20 antibody rituximab as monotherapy (56) or in combination with azathioprine, as reported by some groups (48, 57).

HSCT is not considered to be a standard therapeutic option in patients with GLILD (5). However, HSCT was reported to be applied to some patients with malignancies, refractory autoimmune cytopenias, ILD/GLILD, and/or autoimmune enteropathy, among others in patients with LRBA deficiency (58). As reported by Tesch et al., HSCT in these patients was associated with an increased risk of death in the course of early post-transplant complications, but HSCT survivors achieved remission of disease symptoms (including ILD), in contrast to patients who had not undergone transplantation (55). The overall survival rate of patients undergoing HSCT was 70.8%, and the vast majority (70.6%) didn't require further immunosuppressive treatment (55).

Since only one of our patients was treated with HSCT due to refractory autoimmune cytopenia, we did not feel entitled to enclosing a more extensive discussion or drawing any binding conclusions.

### Strength and Limitations of the Study

To our knowledge, this is the largest study on children with PID-related ILD (mostly CVID patients) published so far, presenting detailed description of clinical, radiological, and histological signs of ILD/GLILD. Additionally, the analysis of the first- and second-line treatments is presented.

However, this study also has some limitations. First of all, it is a retrospective analysis. As a consequence of the study design, there are further drawbacks, including genetic diagnosis in only a few patients and non-uniform assessment and treatment.

It is important to point out that two of the three children with the most advanced lung disease were not able to perform PFT due to young age or intellectual disability. Finally, the type of ILD was not confirmed by surgical lung biopsy in three of the children.

We believe that our study clearly points out the need for developing a uniform and commonly accepted diagnostic and therapeutic algorithm for patients with PID and coexisting ILD. Special attention should be paid to early radiological detection of lung involvement with the use of HRCT. This is supported by our finding that some asymptomatic patients with PID-related ILD may present with advanced structural lung alterations. It also seems necessary to propose a more uniform therapeutic approach and to gather more long-term observations on the relationship between treatment regimen and disease outcomes.

## Conclusions

Our analysis showed that the clinical presentation of PID-related ILD can be diverse, ranging from asymptomatic presentation to life-threatening disease. It should be emphasized that ILD symptoms may precede the diagnosis of PID and that aggressive treatment, especially in patients with a rapid clinical course, provides fast but sometimes incomplete improvement. Although corticosteroids seem to be effective in the treatment of PID-related ILD, even in severe cases, the optimal treatment of PID-related ILD patients is hindered by the lack of clinically proven treatment regimens for these diseases. Hence, there is a need for a well-designed, multicenter study that includes an adequate number of patients, including pediatric population, to determine the most effective treatment strategies and the optimal duration of the therapy.

## Data Availability Statement

The raw data supporting the conclusions of this article will be made available by the authors, without undue reservation.

## Ethics Statement

The studies involving human participants were reviewed and approved by The Bioethics Committee of the Children's Memorial Health Institute in Warsaw. Written informed consent to participate in this study was provided by the participants' legal guardian/next of kin. Written, informed consent was obtained from all individuals AND/OR their parents for the publication of any potentially identifiable images or data included in this article.

## Author Contributions

MPa: conceived the idea for the study. MPa, TB, KG, and KK: study design, coordination and supervision of data collection, data analysis, and manuscript preparation. RL: preparation and assessment of histopathological preparations and manuscript edition. JK: radiological assessment and manuscript preparation. MPr: preparation and assessment histopathological preparations and critical review. BP and KT: contributed to immunologic data collection and critical review. SK, ND-L, KB-S, HD, AP-N, BM, EB, and IW-B: contributed to clinical data collection and critical review. All authors: contributed to the article and approved the submitted version and agree to be accountable for all aspects of the work.

## Conflict of Interest

The authors declare that the research was conducted in the absence of any commercial or financial relationships that could be construed as a potential conflict of interest.
